# Role of information and preparation for improvement of pediatric perioperative care

**DOI:** 10.1111/pan.14419

**Published:** 2022-03-13

**Authors:** Gunilla Lööf, Per‐Arne Lönnqvist

**Affiliations:** ^1^ Paediatric Perioperative Medicine and Intensive Care Karolinska University Hospital Stockholm Sweden; ^2^ Department of Learning, Informatics, Management and Ethics Karolinska Institutet Stockholm Sweden; ^3^ Department of Physiology and Pharmacology Karolinska Institutet Stockholm Sweden

**Keywords:** anesthesia, child, education, information, interactive, preparation, web‐based technology

## Abstract

The perioperative period is a significant and stressful experience that may cause negative consequences in children, both in a short‐term and long‐term perspective. Despite a wide base of evidence stating the importance of adequate preparation to reduce anxiety, improve coping, cooperation and enhance recovery, many children continue to report that they feel unprepared for their perioperative experience. To secure children's right to request and need for preparation, the content, format, and availability of existing programs need to be scrutinized. Preparation programs in perioperative care must change from simply providing information to embracing the importance of children's need to process the information provided in order to learn and understand. Interactive web‐based technology can function as a significant resource for preparation of children for perioperative procedures. By changing perspective from children's need for information to their need for learning and by developing preparation programs including adequate educational principles, web‐based technology can be used to its fullest advantage as a healthcare learning and preparation resource.

## 1 INTRODUCTION

The perioperative period is a significant and memorable event in the life of children. It is a stressful experience, which may cause psychological and behavioral consequences, affect cooperation, treatment, recovery as well as future dealings with medical services.^1^, ^2^

Children have many questions, want to be prepared for the perioperative procedures and be involved, consulted, and heard in relation to their informational needs.^2^, ^3^, ^4^ Access to understandable information and preparation are also recognized as fundamental rights for children in a medical context.^5^ Despite a wide base of evidence stating the importance of adequate preparation to establish understanding, trust, and confidence,^2^, ^3^, ^6^ the content, format, and availability of preparation programs for children prior to the perioperative process varies greatly.^7^ The aim with this educational review is to provide an overview and update of perioperative preparation programs for children, which will hopefully promote a more widespread and adequate use of the currently available options. To enhance the quality of the perioperative process, children's need for an understandable, repetative and continiously accessible preparation need to be secured and ranked in equality to other perioperative preparations. New concepts and programs need to not only focus on *what* information is given, but also to *how* the information is interpreted and understood of each individual child. Interactive, age‐specific web‐based alternatives currently appear to be best suited for this further improvement.

## 2 PREOPERATIVE ANXIETY

Preoperative anxiety is a well described phenomenon affecting up to 60% of children undergoing surgical procedures.^8^, ^9^ Variables such as gender, age and cognitive ability, baseline anxiety, temperament, previous medical experiences, and anxiety of the parent have been identified as predictors of preoperative anxiety.^1^, ^10^ Child temperament and parental anxiety appear to be risk factors for high levels of child anxiety across the perioperative setting, from the preoperative holding area until 2 weeks following surgery.^11^ Elevated levels of preoperative anxiety have been associated with increased postoperative anxiety, pain and analgesic requirements and longer and more complicated recovery.^1^, ^12^ In turn, preoperative anxiety is associated with high incidence of long‐term behavior change in children including separation anxiety, sleep disturbance, temper tantrums, bed‐wetting, eating problems, and delayed recovery.^1^ The long‐term negative behavior changes are of particular concern if they impact negatively on the child's responses to future medical care or interfere with emotional and cognitive development.^13^

Most studies are reporting the incidence of and risk factors for preoperative anxiety in children undergoing anesthesia and surgery.^9^, ^14^ Children's own experiences highlight the need and continued effort to not only focus on the preoperative process but also to reduce stress and anxiety across the entire perioperative period.^2^, ^4^, ^11^, ^15^

## 3 STRATEGIES TO IMPROVE THE PERIOPERATIVE PROCESS IN CHILDREN

To reduce the incidence of perioperative stress and anxiety in children several preventive strategies have been employed and studied. These can broadly be categorized as: pharmacological and non‐pharmacological interventions including: *social, communicative and behavioral, environmental, psychological, and educational interventions* ^6^, ^7^, ^16^ (Table 1).

**Table 1:** Non‐pharmacological strategies to alleviate children's perioperative stress and anxiety

Social interventions	Parental presence
Communicative and behavioral interventions	Healthcare providers behavior and interaction.
	Distractive and nonprocedure‐related talk and humor.
	Medical reinterpretation
Environmental interventions	Transport in a toy car.
	Anesthesia induction rooms.
Psychological interventions	Distractive stimuli
	Hospital clowns
	Smartphones and tablets
	Guided Imagery
	Hypnosis
	Virtual reality
Educational interventions	Preparation programs
	Book and booklets
	Therapeutic play
	Theater orientation
	Multicomponent preparation
	Audiovisual preparation
	Interactive preparation
	Web‐based preparation

### 3.1 Pharmacological interventions

In the past, pharmacological interventions were necessary to provide smooth and safe anesthesia. The main goals were to achieve a general stress reduction, to provide control of autonomic reflexes and to counteract side effects of anesthetic drugs and procedures. With the development of both the practice of anesthesia and anesthetic agents, and the availability of distraction and preparation programs, the need for routine pharmacological preparation of children has decreased markedly.^17^ To avoid the undesirable and adverse pharmacological side‐effects, and to help children to develop appropriate coping strategies to deal with similar situations in the future, the value of non‐pharmacological strategies is increasingly recognized (Table 1). Non‐pharmacological interventions have also been found to be as effective as pharmacological interventions to reduce preoperative anxiety in children.^7^

### 3.2 Non‐pharmacological interventions

#### 3.2.1 Social interventions

Parental presence during the induction of anesthesia is a continuing matter of discussion and is practiced to a varying extent over the world.^7^ Research suggests that parents often prefer to be present during their child's induction of anesthesia and believe that their presence is helpful.^18^ From a psychological point of view, parental presence has been reported as crucial in such a highly stressful and major life event as anesthesia. Parental presence during induction of anesthesia has also been argued as a civil right for both children and parents.^17^ The rationale for allowing parental presence during induction is that the presence of a consistent, responsive, and emphatic caregiver ensures psychological support which eases the child's adaption to the unknown environment. Additionally, parents usually have a better knowledge of the child's responses and preferred coping style and are functioning as important stress‐regulators for children.^18^ Despite conflicting evidence^7^ it thus seems appropriate that parents are provided with opportunities and prerequisites to be present during their child's induction of anesthesia.^18^

#### 3.2.2 Communicative and behavioral interventions

Data representing the relationship between the behavior of healthcare providers and children's distress and coping during the perioperative process indicate that the behavior of the healthcare provider matters and positively or negatively can influence the levels of children's perioperative stress and anxiety.^19^, ^20^ Distracting and nonprocedure‐related talk and humor have shown to reduce children's levels of stress and be significantly positive related to children's coping behaviors, whereas emotion‐focused behavior such as empathy and reinsurance have shown to be positively related to higher levels of children's distress. Medical reinterpretation (i.e., reframing medical equipment and procedures as non‐threatening) has shown to be positively related to children's regulating and coping behaviors when performed by the anesthesiologist and used to reference equipment and procedures within children's immediate environment, but related to increased distress when performed by parents and in the child's immediate environment.^20^ Awareness of these connections among healthcare providers are described as important for management of children's perioperative stress and anxiety.^2^, ^21^

#### 3.2.3 Environmental interventions

Although the entire perioperative process is stressful for children, the entrance to the operating room and the anesthesia induction are ranked as the most stressful parts.^2^, ^8^, ^15^

Several environmental interventions have been considered to reduce children's anxiety during these stages of the perioperative process. Transport from the ward to the OR in a ride‐on toy car have shown to relieve preoperative anxiety in preschool children to a comparable degree as midazolam.^22^ The use of anesthesia induction rooms has proved to be more appropriate regarding several assessed anxiety measures (momentary anxiety, environmental anxiety, and electrodermal activity) compared with anesthesia induction performed in the operating room.^23^

#### 3.2.4 Psychological interventions

Distraction is commonly used, and in a large number of publications proved as effective to reduce children's stress and anxiety by redirecting attention from a perceived threatening to a relaxing or entertaining stimuli.^7^ The spectra of distractive stimuli evaluated with variated positive relations to children's reduction of perioperative distress include among others; hospital clowns,^24^, ^25^ smartphone or tablet‐based distraction (e.g., video games, smartphone apps, or video clips),^26^, ^27^, ^28^, ^29^, ^30^ guided imagery,^31^ hypnosis^32^ and virtual reality.^33^, ^34^, ^35^

Although distraction undoubtedly is associated with positive effects, a clear distinction must be drawn between the use of distractive stimuli and educational preparation programs to reduce perioperative stress and anxiety in children. While the focus for distractive stimuli is to decrease distress by redirecting children's attention from fearful events,^7^ educational preparation programs aim to reduce stress and anxiety by increasing children's understanding and learning of the perioperative procedures.^36^, ^37^, ^38^

#### 3.2.5 Educational interventions

A wide base of evidence supports the role of educational preparation programs to reduce children's perioperative stress and anxiety, improve coping and cooperation, enhance postoperative recovery as well as short‐ and long‐term psychological and behavioral consequences.^36^, ^37^, ^38^ Educational preparation material have shown to significantly reduce children's worries during the perioperative process compared with distraction in terms of entertaining material, as well as to be the most appropriate and effective method for children to learn coping strategies.^16^

## 4 EDUCATIONAL PREPARATION OF CHILDREN FOR PERIOPERATIVE PROCEDURES

The way that children view and understand the perioperative procedure will influence how they respond to it. Information and preparation help children shape that lens which in turn shapes the experience, as well as the affective and behavioral responses.^39^ Preparation of children regarding the perioperative process thus is of great importance but represents challenges related to the distinct characteristics of children and requires specialized expertise.^37^

Comprehension of the perioperative process is a decisive factor for children's expressions of confidence or fearfulness during the perioperative period.^2^ Proper preparation helps children to establish trust and safety by learning and understanding about procedures and generate accurate expectations of what will be experienced.^6^ This is important since children of today may have a distorted picture of sickness and hospitalization.^40^ Providing children with reality‐based information help them regulate their expectations and alleviate their fears.^6^ Preparation is also important to increase children's cooperation and reduce short‐term and long‐term negative behavioral consequences, to help children gain trust and confidence in healthcare providers, to deal with what has happened and to better cope with future medical care and treatment.^16^, ^36^, ^37^

Preparation of children should make use of the following strategies: information provision, modeling (observation of targeted skills), and coping.^41^ According to social learning theories^42^ certain behaviors can be learned from and reproduced, under similar conditions, by observing the actions performed by others. Modeling can be a very effective tool to provide information, teach effective behaviors, and to reinforce self‐efficacy and may be even more effective if it portrays children or symbolic models of the same age as the user that increase the identification with the model.^6^, ^42^

Furthermore, adequate preparation of children for perioperative procedures requires understanding of how children understand and learn from the information given. Of relevance is not only *what* information is given to the child, but also *how* the information is interpreted and understood. Preparation of children for perioperative procedures so far mostly has focused on *the what‐aspect* (the content) achieved by a one‐way distribution of information from the healthcare providers. Only receiving information does not guarantee that the child has learned and understood.^38^ Learning is a multifaceted phenomenon that requires processing of information cognitively, emotionally, and socially through testing and practical actions.^43^ Children need time and tools to process the information given to learn and fully understand. For optimal outcome of the preparation of children for the perioperative process, the *how‐aspect* (the process) of children's understanding and learning has to be fully recognized.^38^

**Figure 1:**
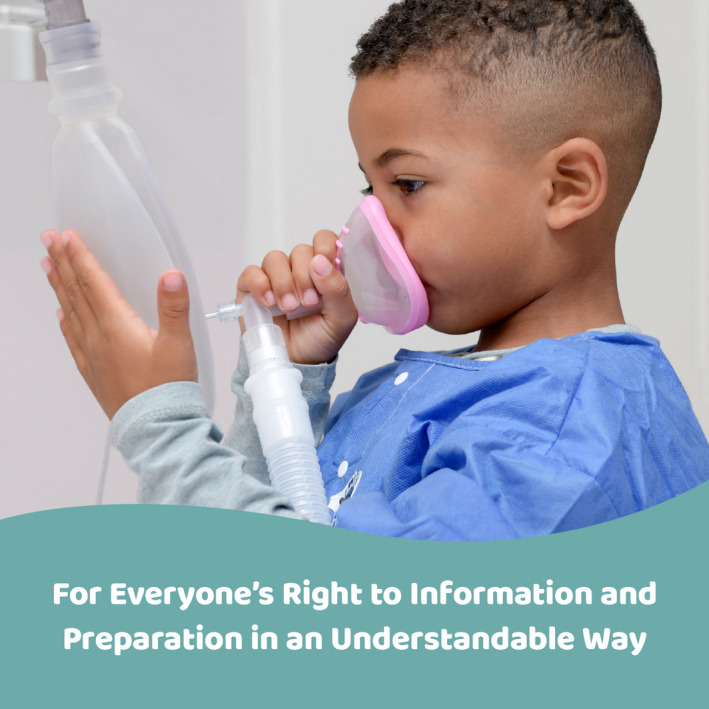
Children have need for, and the rights to, understandable information and preparation for the perioperative process. Photographer: Christin Philipson

Careful consideration thus needs to be given not only to what information children should receive (content), but also to how the information should be conveyed (format) and when it should be provided (timing).^6^, ^38^ Children represents a spectrum of ages whose need and ability to process and learn from given information differs widely. It is important that the information is designed and conveyed in a manner which is consistent with children's cognitive and psychological developmental levels both content‐wise, technically, pedagogically, and linguistically^6^ (Figure 1). In this context, it is important to understand that children who are highly stressed may be functioning at lower cognitive levels than they otherwise would.^6^ Because it may be difficult for children to translate procedural information into a clear set of expectations about what they will experience, the information should be concrete and a combination of both procedural (*what will be done*) and sensorial information (*what will be experienced*).^6^ At this point it is important to highlight that the preparations take into due consideration the fact that it needs to be adjusted according to the age of the child and the special concerns involved with handling children with various types of cognitive limitations.^44^, ^45^, ^46^

Children express both the importance of different formats for delivering the information and repeated opportunities to prepare as crucial for their preparation and understanding.

The perioperative process is a period of continuing activity. Even though children are prepared for the expected procedures they most likely will be exposed to new, unexpected experiences highlighting the importance of not restricting children's need for information and preparation to a single event. A continuous process signified by individualized adjustments and flexibility emphasized during all the perioperative phases has to be provided.^2^, ^4^ Information exclusively provided bedside on arrival to the hospital may be seen as convenient but is clearly suboptimal. A short time frame combined with high levels of stress will limit children's ability to process and learn from the information given. On the other hand, information received prior to the day of surgery reinforced on admission to the hospital both allows children to process the information given and prepare questions to the healthcare providers.^2^, ^4^, ^38^

Children undergoing repeated perioperative procedures also require repeated preparation.^3^, ^6^ Previous experiences may trigger negative cycles, whereby expectations are worse than the reality. This might lead to poorer outcomes, thus potentially reinforcing the negative beliefs.^39^ Children's capability to process and understand the information given also changes with their cognitive development. Preparation of this group of children thereby needs to be adapted both to their previous experiences and their current cognitive developmental level.^3^, ^6^ In some cases, alternative psychological preparation programs such as extensive individualized coping skills training based on the particulars child's experience have to be considered.^47^

## 5 PREPARATION PROGRAMS IN PERIOPERATIVE CARE

The development of perioperative preparation programs for children have evolved over the last decades. The current available preparations programs include *book and booklets*, *therapeutic play and theater orientation*, *multicomponent preparation*, *audiovisual, and interactive interventions* and *web‐based preparation programs*.^7^, ^36^

### 5.1 Book and booklets

The effectiveness of educational books and booklets containing anesthesia and/or surgery related information on children's pre and postoperative behavior is unclear. Some studies have shown a significant reduction in preoperative anxiety levels in pre‐school and school‐aged who received educational books compared with children who received face‐to‐face verbal preparation.^48^ Levels of literacy can also hamper leaflet‐based education, restricting access for those who may need the information most.^49^

### 5.2 Therapeutic play and theater orientation

The use of therapeutic play as a single method or combined with theater orientation has showed to reduce children's preoperative anxiety and negative emotional manifestations during mask induction of anesthesia.^48^, ^50^ A brief, targeted child life preparation session also have been shown to be effective in reducing preoperative anxiety during IV placement in the operating room prior to intravenous induction of anesthesia in children.^51^ However, despite the positive effects described, empirical evidence found on the effectiveness of therapeutic play interventions in children's perioperative anxiety, negative behaviors, and postoperative pain is inconclusive. This may be a result of the heterogeneity of the various study designs, outcome measurements and timing at which the outcomes were measured. Hence, studies with high methodologic quality are required to establish the effect of therapeutic play during children's perioperative procedures.^50^

### 5.3 Multicomponent preparation

Several studies declare that multicomponent preparation is effective in reducing both child and parental anxiety in the holding area and during anesthesia induction, yet no significance has been found in children's postoperative compliance.^48^ Family centered multicomponent behavioral preparation program has been proved to be effective for children's anxiety, incidence of emergency delirium, requirements of analgesia in the recovery room and discharge from the hospital.^52^ Exposure to the anesthesia mask and parental use of distraction have been found as the most critical components for reducing anxiety in family centered multicomponent behavioral preparation program.^53^

### 5.4 Audiovisual and interactive interventions

In regards to preparation of children with audiovisual interventions, multi‐faceted preparation programs including videos and interactive components are most effective in reducing preoperative anxiety, postoperative pain, negative behaviors and increase compliance during the perioperative process.^26^ Compared to verbal information, preparation with interactive computer packages have shown to be more effective both in increasing cooperation during the anesthesia induction and to reduce immediate postoperative negative behavioral changes in children undergoing dental surgery.^54^ All technology‐based interventions are able to contribute to a reduction of preoperative anxiety, but the effects are most consistent for the use of tablets and handheld devices.^55^

Play constitutes an important part of children's learning and understanding. Interactivity is an important part of children's play, which enables children to learn with all their senses to understand the situations encountered.^56^ Intrinsic motivation refers to carrying out an activity because the activity itself is enjoyable or interesting. Carrying out intrinsic motivated activities does not only contribute to children's wellbeing but can also enhance the acceptability of a particular message and is connected to profound learning, better learning results, more creativity, and longer sustaining in an activity.^57^

A computer game can provide information about a rather frightening subject like a surgical procedure in a less intimidating way. In order to help children to prepare with games, it is necessary to assure that the games provided prepare them for the medical procedures ahead even though games as a distractive tool has found to reduce anxiety significantly compared with children who did not play any game during anesthesia induction.^58^ Games, including preparation about the perioperative process, have been proven to significantly reduce anxiety compared to children's baseline before playing the game. Children also had a significant lower anxiety score playing a distraction game compared to receiving only verbal information in a preanesthetic clinic.^59^

Tablet and hand‐held devices with interactive components represent the strategy with the most encouraging evidence for preoperative preparation of children, while video preparation alone may provide sufficient information to manage preoperative anxiety in parents.^55^ The use of an educational video for parents also has proved to be an effective tool to increase parental knowledge and participation that in turn led to significant reduction in children's postoperative pain.^60^

### 5.5 Web‐based preparation

Despite all benefits of educational interventions available, children continue to report that they have unanswered questions and have to experience the perioperative process unprepared.^2^, ^3^, ^61^ Researchers have looked to computer technology to aid the preparative and educational process for children prior to the perioperative process.

WebTIPS (Web‐based Tailored Intervention for Preparation of Parents and children undergoing Surgery)^62^, ^63^ comprises education, skills training, games and coping skills to prepare children for perioperative procedures. WebTIPS also includes various modalities for parental preparation including anxiety management and training in relaxation and distractive techniques. WebTIPS have been shown to result in improved immediate behavioral outcomes, including decreased preoperative anxiety in parents and children and decreased emergence delirium in children.

I‐PPP (Internet‐delivered Preoperative Preparation Program) is a comprehensive, interactive preparation program designed for children aged 3–8 years undergoing day surgery, and their parents.^64^ The I‐PPP includes education about the process of day surgery and anesthesia, identification of common experiences and emotions, behavioral training and practice of skills. Compared to regular treatment, I‐PPP have been shown to reduce anxiety an increase compliance in children during induction of anesthesia, but not necessarily for their parents.^65^

The Anesthesia Web (www.anaesthesiaweb.org) represents a comprehensive, age‐specific, interactive, multimedia web‐based, non‐profit preparation program to prepare and educate children and parents prior to perioperative procedures. The Anesthesia Web includes videos, cartoons, podcasts, and FAQ. Children gets the opportunity to meet others with similar experiences, play games, do crafts and paint and play in their own operating room. The Anesthesia Web is fully available in English and Swedish. Information for parents is available in 32 different languages. The Anesthesia Web has open access and can, as all the content on the site is generally applicable, be used irrespective of local protocols. The Anesthesia Web has been developed by a multidisciplinary team together with children of all ages, all with various experiences of healthcare, anesthesia, and surgery.

Based on an educational framework of children's learning, the Anesthesia Web has been developed to be accessible to all children in terms of content, technology, pedagogy, and language.^38^ The child component makes use of preparation strategies such as information provision, modeling, play, and coping skills training. The use of explicit modeling videos allows teaching of specific procedures, behavior, and coping skills. Analysis based on a pedagogical framework have proved the Anesthesia Web to be an effective tool for children's preparation for and learning of perioperative procedures.^38^, ^66^ Furthermore, the Anesthesia Web also have shown to significantly increase knowledge and understanding about the perioperative procedure both in children and parents, compared to when the information was given with conventional brochure material.^67^

## 6 DISCUSSION

Reports are indicating the limited time healthcare providers today spend with the families before perioperative procedures and decreasing numbers of children having access to and attending preparation programs.^1^, ^68^ The move towards cost and time efficiency, an increasing shift towards outpatient surgery, as well as a result of covid‐associated regulations, results in that children's opportunities to prepare and learn about the perioperative process, as well as their opportunities for follow‐up of questions and concerns are considerably decreasing.

With the proliferation of technology and the ubiquitous presence of internet and mobile media, web‐based preparation offers a powerful tool to secure the information and preparation of children and families for perioperative procedures.^55^ Children and most caregivers of today have the unique experience of being surrounded by digital media from a very young age. Children themselves also declare Internet as their primary source of information ahead of contact with healthcare and treatment.^2^ Computer technology is flexible, can be tailored to the individuals’ information needs, provides ongoing educational support and promotes learning. In addition, the availability of a website allows the user to access the program in the comfort of their own homes, as many times and when is convenient for them during the perioperative process.^38^, ^49^, ^66^

Today, there is an increasing development and availability of web‐based programs for children in pediatric care. The content and design of web‐based preparation programs require careful consideration. Web‐based information can be interactive, and patient centered, but if it is not used with consideration of children's processes of understanding and learning, it might work only as another information provider.^38^, ^66^

Computer programs designed to educate children must be built on a robust theoretical framework, or risk failure, in the same way as any other educational intervention. Encouraging active learning is central to multimedia education so promoting engagement through interactivity is a key. However, obtaining a balance between being sufficiently sophisticated to maintain interest, while allowing easy widespread usage can be challenging.^38^, ^66^ Since internet sites are largely unregulated and of variable quality, it is both essential that educational web‐based interventions are evidence‐based and rigorously evaluated and that families are provided with references on high‐quality web‐based sources of information.^49^

Web‐based preparation represents an additive intervention aimed to improve the perioperative care we provide our children and families. When the children arrive to the perioperative procedures, more time will be available for individual interactions since the preparation has already started. Furthermore, communication and report between children and healthcare providers can be enhanced through discussions about the specific content on the website. It can give children a sense of “shared experience” which can promote empowerment with positive psychological and physical consequences.

## 7 CONCLUSION

Evidence reporting on children's perioperative anxiety and lack of preparation call for extensive changes. Preparation of children for the perioperative process often seems to be based on clinical intuition and based on delivery of information rather than on empirical knowledge and mechanisms associated with how children perceive, process, and learn from such information. To secure children's rights to request and need for preparation, we would argue it is time to scrutinize the content, format, and distribution of the existing preparation programs and consider new approaches when preparing children for the perioperative process. *First,* preparation of children must be performed and ranked in equality to other perioperative preparations. *Second,* preparation programs must change from simply providing information to embracing the importance of children's need to process the information provided in order to learn, understand and be prepared. By changing perspective from children's need for information to their need for learning, and by developing targeted preparation programs including adequate educational principles, web‐based technology can be used to its fullest advantage as a healthcare learning and preparation resource.

### 7.1 Reflective questions

1. How can information and preparation of children be performed and ranked in equality to other perioperative preparations?
2. How can preparation of children shift from only focus on *what* information is given to also include *how* the information is interpreted and understood by children?
3. How can the preparation be provided to secure children's need to process the information given in order to learn, understand and be prepared?

## Data Availability

Data sharing is not applicable to this article as no new data were created or analyzed in this study.
